# Novel genes exhibiting DNA methylation alterations in Korean patients with chronic lymphocytic leukaemia: a methyl-CpG-binding domain sequencing study

**DOI:** 10.1038/s41598-020-57919-6

**Published:** 2020-01-23

**Authors:** Miyoung Kim, Eunyup Lee, Dae Young Zang, Hyo Jung Kim, Ho Young Kim, Boram Han, Han-Sung Kim, Hee Jung Kang, Seungwoo Hwang, Young Kyung Lee

**Affiliations:** 1 Department of Laboratory Medicine, Hallym University Sacred Heart Hospital, Anyang, Republic of Korea; 2 Department of Internal Medicine, Hallym University Sacred Heart Hospital, Anyang, Republic of Korea; 3 Korean Bioinformation Center, Korea Research Institute of Bioscience and Biotechnology, Daejeon, Republic of Korea

**Keywords:** Chronic lymphocytic leukaemia, Genetics research

## Abstract

Chronic lymphocytic leukaemia (CLL) exhibits differences between Asians and Caucasians in terms of incidence rate, age at onset, immunophenotype, and genetic profile. We performed genome-wide methylation profiling of CLL in an Asian cohort for the first time. Eight Korean patients without somatic immunoglobulin heavy chain gene hypermutations underwent methyl-CpG-binding domain sequencing (MBD-seq), as did five control subjects. Gene Ontology, pathway analysis, and network-based prioritization of differentially methylated genes were also performed. More regions were hypomethylated (2,062 windows) than were hypermethylated (777 windows). Promoters contained the highest proportion of differentially methylated regions (0.08%), while distal intergenic and intron regions contained the largest number of differentially methylated regions. Protein-coding genes were the most abundant, followed by long noncoding and short noncoding genes. The most significantly over-represented signalling pathways in the differentially methylated gene list included immune/cancer-related pathways and B-cell receptor signalling. Among the top 10 hub genes identified via network-based prioritization, four (*UBC*, *GRB2*, *CREBBP*, and *GAB2*) had no known relevance to CLL, while the other six (*STAT3*, *PTPN6*, *SYK*, *STAT5B*, *XPO1*, and *ABL1*) have previously been linked to CLL in Caucasians. As such, our analysis identified four novel candidate genes of potential significance to Asian patients with CLL.

## Introduction

Chronic lymphocytic leukaemia (CLL) is characterized by the co-expression of CD5 and CD23 in monomorphic small B-cells; it is the most common leukaemia among adults in Western countries, especially the elderly^1^. CLL among Asians differs from that among Caucasians in terms of the incidence rate, median age at onset, phenotype, and the genetic profile. The incidence rates per 100,000 person-years in Korea and Japan are 0.04 and 0.48, respectively, but the rate is 3.83 in Western countries^2^. The median age at initial CLL diagnosis is 61 and 70 years among Asians and Caucasians, respectively^2^. A single-institution study in South Korea found that 56% of patients had an atypical immunophenotype with high frequencies of FMC7 positivity and strong CD22 positivity^3^. A Chinese study showed that *TP53* mutations are more common in Chinese patients with CLL than in Caucasian patients, whereas *SF3B1* mutations are less common^4^. Furthermore, a Korean study found that the frequencies of mutations in *ATM, TP53, KLHL6, BCOR*, and *CDKN2A* tend to be higher in Koreans than in Caucasians, while those in *SF3B1, NOTCH1, CHD2*, and *POT1* tend to be lower^2^.

DNA methylation directly impacts human genome function, and multiple studies have demonstrated the existence of aberrant epigenetic changes that play important roles in tumour initiation and progression in Western patients with CLL^5–8^. Recent advances in high-throughput techniques have enabled genome-wide methylation profiling in Caucasians with CLL. For example, an array study identified methylation in seven known or candidate tumour suppressor genes (including *VHL, ABI3*, and *IGSF4*) as well as eight unmethylated genes involved in cell proliferation and tumour progression (including *ADORA3* and *PRF1*) in Swedes with immunoglobulin heavy chain gene variable region (*IGHV*)-unmutated CLL^9^. Another study of the same cohort found 2,239 CpG sites that were differentially methylated in *IGHV*-mutated and unmutated patients; DNA methylation over time was relatively stable, implying that aberrant methylation is an early leukaemogenic event^10^. Another Spanish study of 139 patients that included in-depth interrogation using whole-genome bisulfite sequencing showed that *IGHV*-mutated and -unmutated CLL had differing DNA methylomes that represented epigenetic imprints from distinct normal B-cell subpopulations^11^. Additionally, an American cohort study using reduced-representation bisulfite sequencing showed that CLL cells consistently displayed higher intra-sample variability in DNA methylation patterns across the genome, implying that disordered methylation is akin to genetic instability, thereby enhancing the ability of cancer cells to follow superior evolutionary trajectories^12^.

Methyl-CpG binding domain (MBD) sequencing (MBD-seq), a next-generation sequencing technique for genome-wide methylation profiling, sequences the DNA captured by the MBD^13^. MBD-seq is an affinity enrichment-based method in which methyl-CpG binding proteins link to methylated CpGs via the MBD. This technique has several advantages; for example, since DNA methylation occurs primarily within CpG dinucleotides (which represent approximately 1% of the genome), MBD-seq is efficient as it comprehensively interrogates only regions of relevance^14^. Furthermore, MBD-seq does not show restriction enzyme-dependent bias toward CpG-rich regions, resulting in greater genome-wide coverage (61%) than that using reduced-representation bisulfite sequencing (12%)^15^. Compared to array platforms, the genome-wide coverage of MBD-seq is also not restricted to the fixed array content. MBD-seq use has been increasing owing to the aforementioned reasons; however, only one MBD-seq-based study of CLL has been published to date^16^. That study showed that 40% and 60% of hypermethylated and hypomethylated genes, respectively, were mapped to noncoding RNAs. It was also observed that the major repetitive elements (such as the short and long interspersed elements) have a high percentage of differentially methylated regions in *IGHV*-mutated subgroups compared to normal controls.

In contrast to Caucasians, from whom abundant data are available, the methylation profiles of CLL in Asians has never been reported. Hence, we aimed to elucidate the role of aberrant methylation in the pathogenesis of Asian CLL for the first time, and to investigate the differences in methylation patterns between Asian and Caucasian patients with CLL. We also sought to identify novel candidate genes with potentially high functional relevance in CLL using protein-protein and protein-DNA network-based approaches that link the differentially methylated genes (DMGs) to known CLL-related genes.

## Results

### Genome-wide distribution of differentially methylated regions

**Figure 1:**
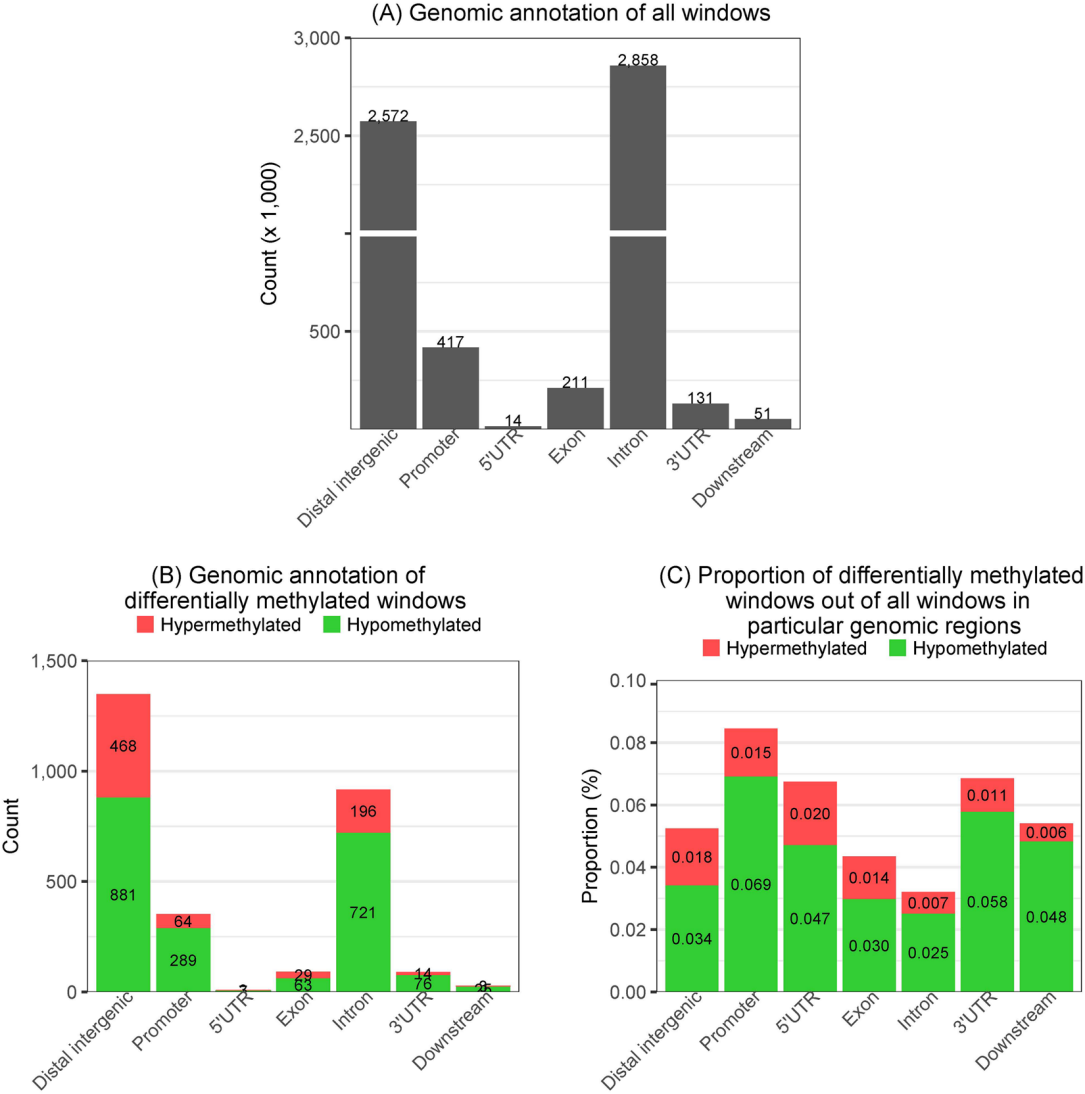
Genomic annotations of differentially methylated regions and their relative proportions with respect to background occurrences in the human genome. **(A)** Genomic annotation of all 250 nucleotide-long windows along the human genome. **(B)** Genomic annotations of the differentially methylated windows. **(C)** Proportion of differentially methylated windows out of all windows in particular genomic regions. UTR, untranslated region.

Our approach using MBD-seq enabled a comprehensive genome-wide interrogation of differentially methylated regions in CLL. Stringent criteria (false discovery rate <0.01) resulted in a total of 2,839 windows of 250 nucleotides that were differentially methylated between the CLL and normal groups. There were more hypomethylated regions (2,062 windows) than hypermethylated ones (777 windows). The genomic annotations of all windows created by binning the human genome into adjacent 250-nucleotide windows are shown in Fig. 1A; introns and distal intergenic regions constituted most (86%) of the human genome, followed by promoter regions (7%). The genomic annotations of the differentially methylated regions are shown in Fig. 1B; they most frequently overlapped with distal intergenic regions, followed by introns and promoters. Hypomethylation was more prevalent than hypermethylation regardless of the type of annotation. The proportions of the differentially methylated regions were obtained by dividing the numbers in Fig. 1B by those in Fig. 1A; this revealed that promoters contained the highest proportion of the differentially methylated regions (0.08%) and were thus the preferential targets of differential methylation in CLL (Fig. 1C). Nevertheless, distal intergenic and intron regions contained the largest numbers of differentially methylated regions (Fig. 1B), highlighting the importance of differential methylation in these regions.

The proportions in Fig. 1C were tested with the two-tailed Fisher’s exact test (Table 1). Promoters and introns were the most significant; differential methylation was observed more often in promoters than would be expected by chance and less often in introns. Distal intergenic regions were the next significant genomic annotation and showed enrichment with differential methylation.

**Table 1:** The extent of association between genomic annotation and differential methylation as tested by two-tailed Fisher’s exact test.

Genomic annotation	P-value	Odds ratio	95% confidence interval
Promoter	<2.2e-16	1.987	1.772–2.222
Intron	<2.2e-16	0.567	0.524–0.614
Distal intergenic	5.39e-12	1.297	1.204–1.397
3′UTR	0.000178	1.528	1.225–1.886
5′UTR	0.2395	1.488	0.713–2.742
Downstream	0.3491	1.195	0.793–1.731
Exon	0.7550	0.958	0.770–1.180

**Figure 2:**
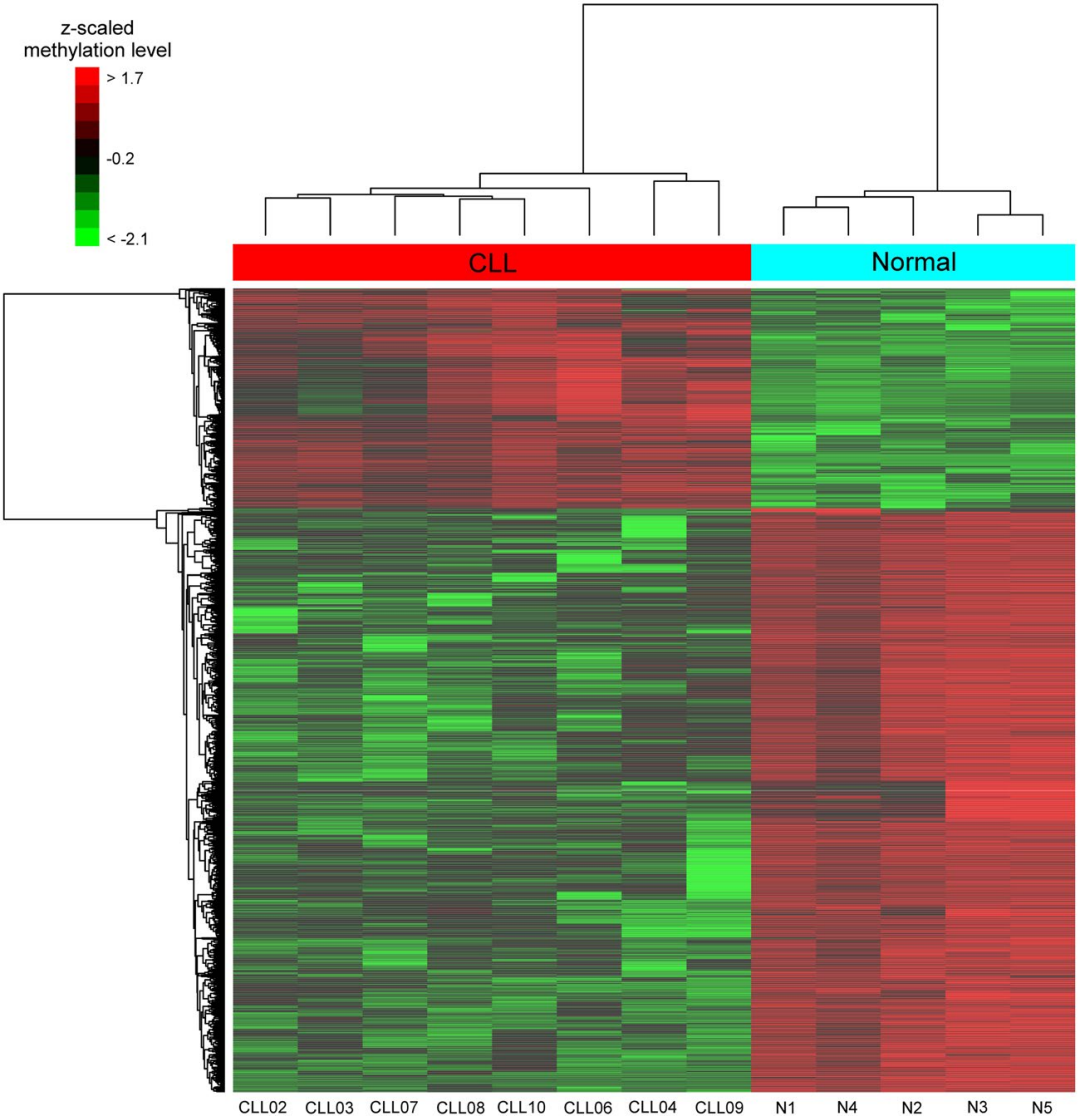
Hierarchical clustering of samples reflecting the profiles of normal and chronic lymphocytic leukaemia (CLL) samples regarding differentially methylated regions. Supervised hierarchical clustering correctly separates the CLL and normal (N) groups. The colour intensity is scaled within each row so that the highest methylation value corresponds to bright red and the lowest to bright green.

When visualized as a heatmap (Fig. 2), the differential methylation profiles indicated more hypomethylated regions than hypermethylated ones. Moreover, the CLL and normal groups were correctly separated by supervised hierarchical clustering with the methylation profile of the differentially methylated regions, as expected.

### DMGs

The list of DMGs (i.e., genes that overlapped with differentially methylated regions) is provided as Supplementary Data S1; there were 1,507 DMGs (1,241 hypomethylated and 315 hypermethylated). Rarely, some of the DMGs showed both hypomethylation and hypermethylation in their genic regions (48 genes; 3%). For brevity, the top 40 hypermethylated and hypomethylated genes are shown in Supplementary Tables 1 and 2, respectively.

**Figure 3:**
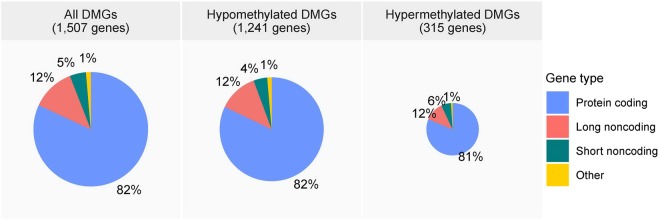
Gene type classification of differentially methylated genes (DMGs).

The DMGs were classified with respect to gene type (Fig. 3). Protein-coding genes were the most abundant, followed by long noncoding genes such as long intronic noncoding RNAs, short noncoding genes such as microRNAs and small nucleolar RNAs, and others such as pseudogenes and unannotated nucleotides. Their proportions in the hypomethylated and hypermethylated DMG sets were similar.

### Gene ontology (GO) biological processes and pathways over-represented in the DMG list

Functional enrichment analyses of the DMG lists identified a number of significantly over-represented GO terms and pathways; up to 30 categories were selected for each condition to examine functional categories specific to either hyper- or hypomethylation.

**Figure 4:**
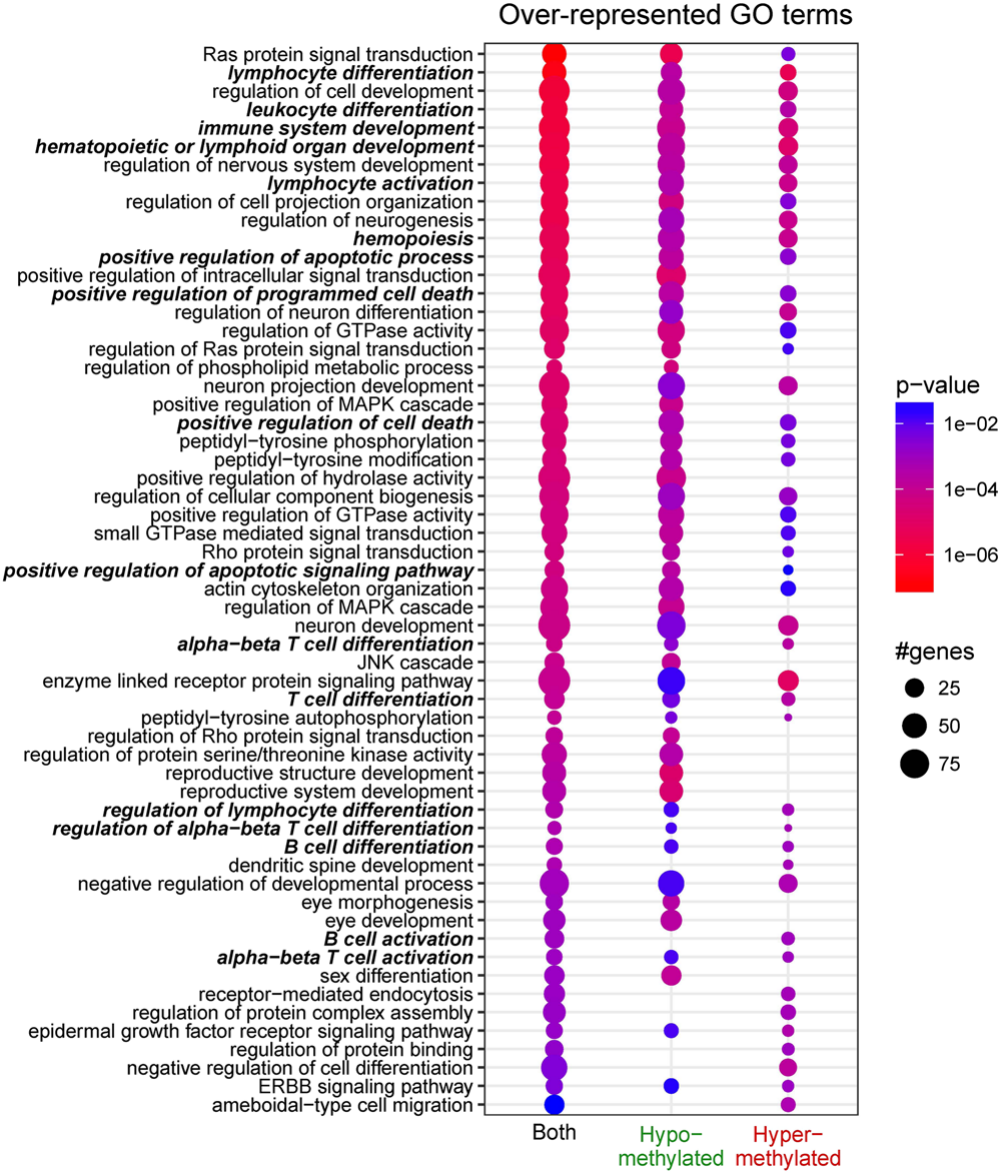
Most significantly over-represented Gene Ontology (GO) terms analysed with respect to differential methylation. A maximum of 30 GO terms were selected for each track; the dot plot shows their p-values according to the displayed colour code. The statistical significance of a GO term increases with a redder tint. The number of differentially methylated genes (DMGs) belonging to a GO term increases with its size. The absence of a dot denotes insignificant over-representation of the corresponding GO terms under that particular condition. GO terms directly related to immune processes and cancer are shown in bold-type.

Numerous immune processes and cancer-related GO terms were over-represented in both the hypo- and hypermethylated gene lists (Fig. 4, highlighted in bold), with the former being more prevalent.

With respect to genomic annotation, most GO terms were over-represented in the genes that showed differential methylation in introns (Supplementary Fig. 1) owing to the presence of many differentially methylated regions. Some immune- and cancer-related GO terms were also over-represented in the genes that showed differential methylation in promoters and exons.

**Figure 5:**
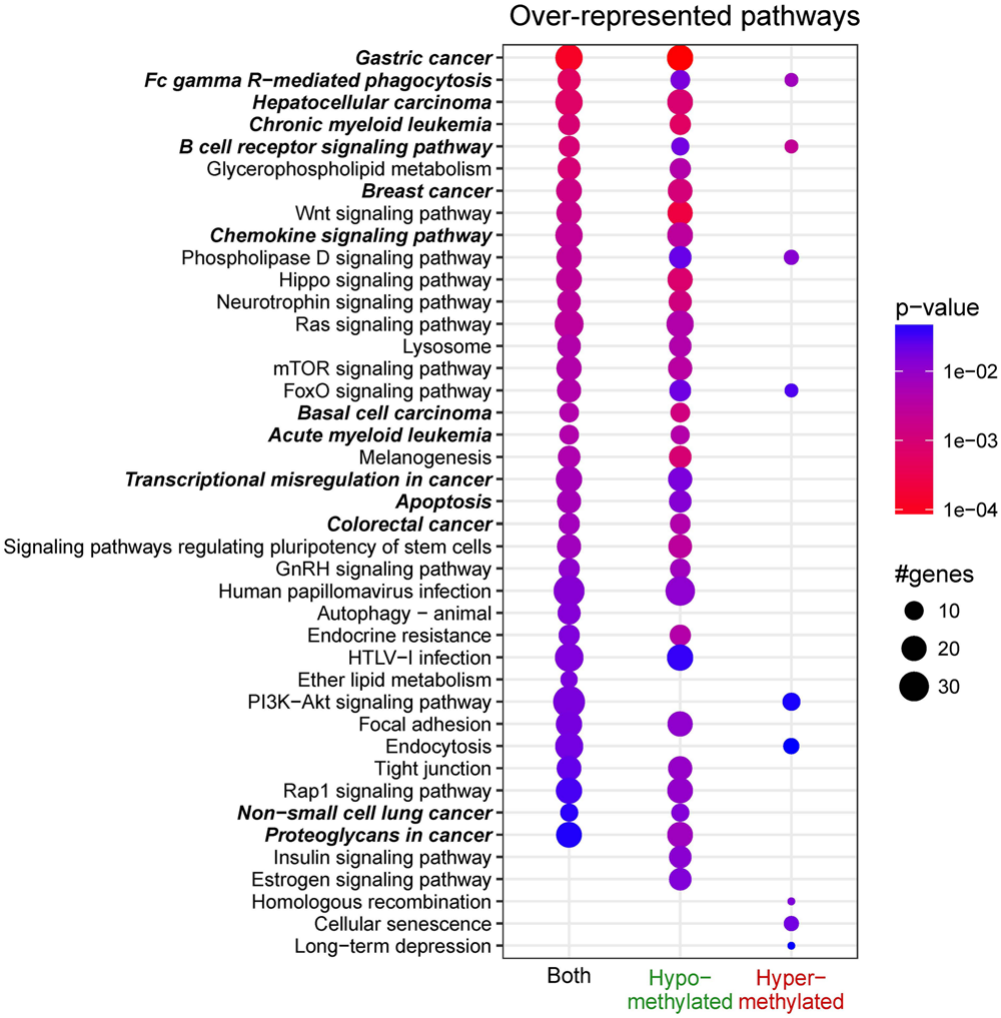
Most significantly over-represented pathways analysed with respect differential methylation. The plot was prepared in the same manner as in Fig. 4.

Over-represented pathways also included those that are directly related to immune processes and cancer (Fig. 5; the Kyoto Encyclopaedia of Genes and Genomes [KEGG] pathway database does not include CLL-related content). Overall, most pathways were over-represented in the gene lists that showed hypomethylation (Fig. 5) or differential methylation of introns (Supplementary Fig. 2). Some immune- and cancer-related pathways were also over-represented in the gene lists that showed differential methylation of promoters and exons.

**Figure 6:**
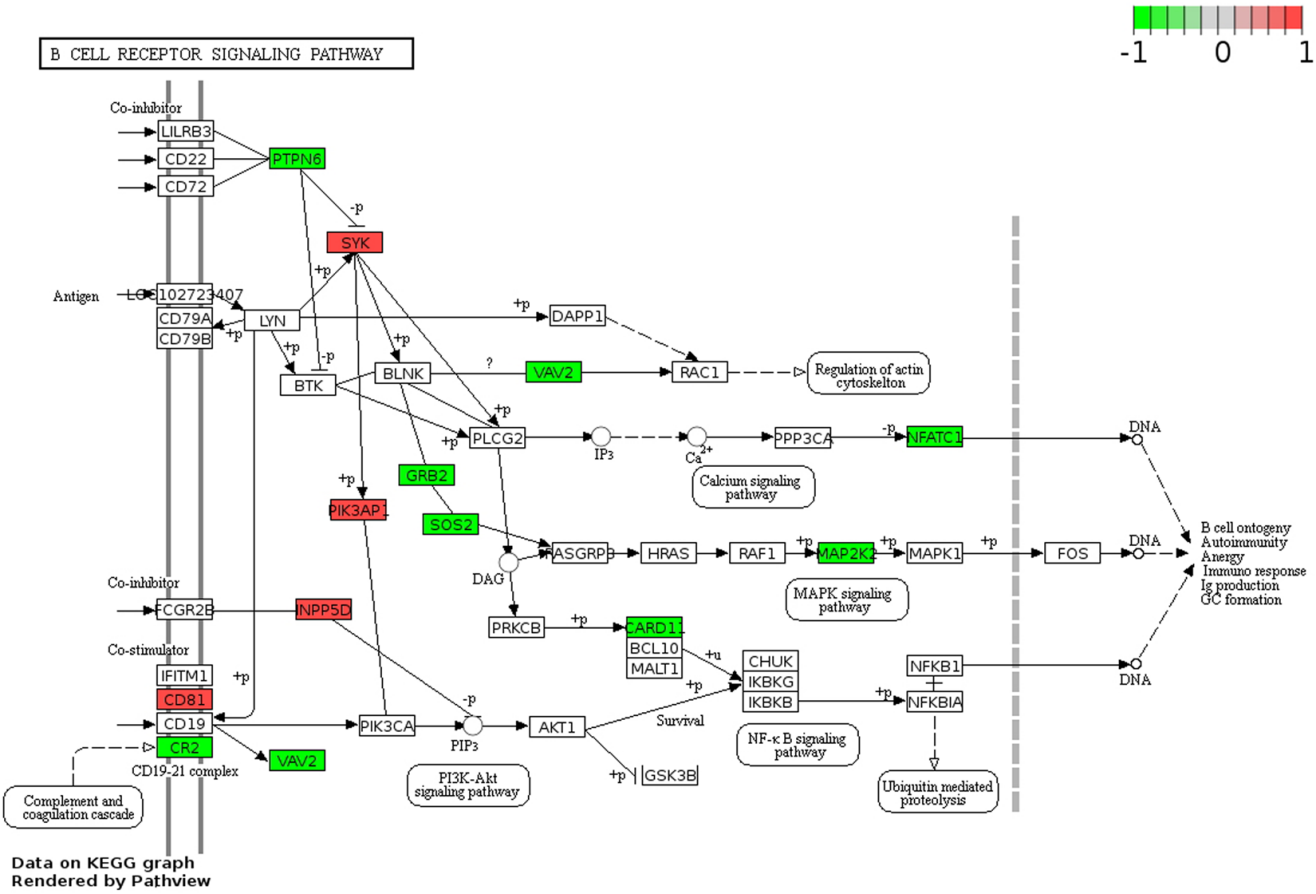
Differential methylation of genes in the B-cell receptor signalling pathway. Hypo- and hypermethylated genes are indicated in green and red, respectively.

The B-cell receptor signalling pathway, which plays a crucial role in the pathogenesis of CLL and is a therapeutic target, was the most significantly over-represented (Fig. 5). The DMGs in this pathway are shown in Fig. 6.

### Network-based prioritization of DMGs

**Figure 7:**
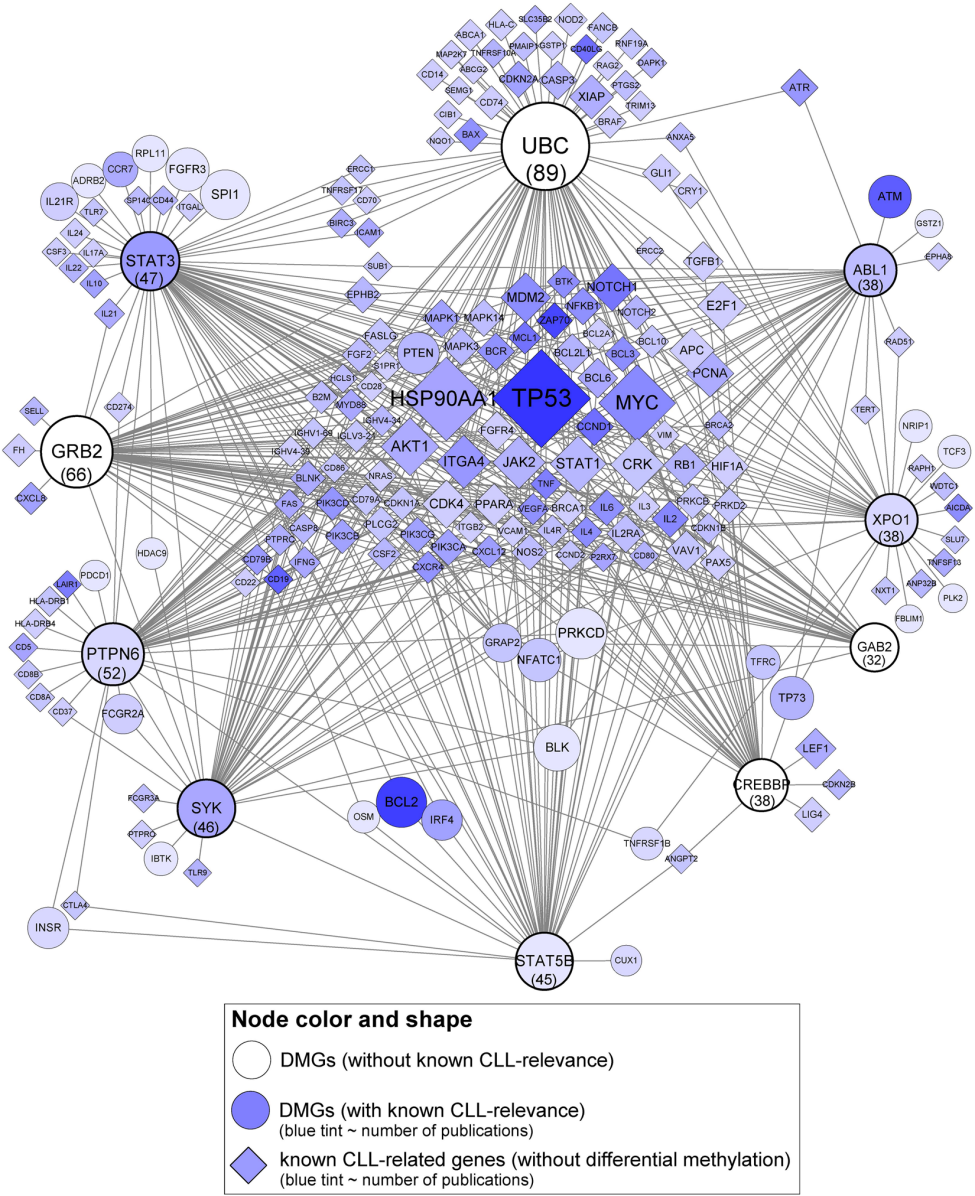
Interaction subnetwork between the top 10 hub differentially methylated genes (DMGs) and known chronic lymphocytic leukaemia (CLL)-related genes. The top 10 hub DMGs are shown along the periphery of the network. Numbers in parentheses denote the node degree of the hub DMGs; the node size is scaled by its node degree.

We performed a network-based prioritization of the DMGs based on the assumption that a DMG that interacts with many known CLL-related genes is also likely to be functionally relevant to CLL itself. A network of 1,094 nodes and 3,304 edges was obtained by linking DMGs to known CLL-related genes by protein-protein and protein-DNA interactions. For easier navigation, we first selected the 10 hub DMGs with the strongest links to known CLL-related genes, then extracted a subnetwork that consisted of the top 10 hub DMGs and their known CLL-related gene interactors. The subnetwork comprised 203 nodes and 512 edges (Fig. 7).

The hub DMGs have the potential to influence or be influenced by the activities of their interacting partners; hence, differential methylation of the hub DMGs may have functional implications in CLL. Among the 10 hub DMGs revealed, six (*STAT3*, *PTPN6*, *SYK*, *STAT5B*, *XPO1*, and *ABL1*) had known CLL relevance (blue tint), whereas the remaining four (*UBC*, *GRB2*, *CREBBP*, and *GAB2*) did not. The former corroborated known data, while the latter demonstrates a novel finding.

## Discussion

Our MBD-seq and subsequent bioinformatics analysis showed that Korean CLL shares similar features with Caucasian CLL in terms of frequent hypomethylation in intragenic regions^11,17^ and aberrant methylation in pathways related to B-cell development, immune processes, and cancer^17,18^. The network analysis identified 10 hub DMGs, 4 of which (*UBC*, *GRB2*, *CREBBP*, and *GAB2*) were reported as being CLL-related for the first time in our study, suggesting potential methylome differences between Korean and Western CLLs.

We identified a total of 2,839 differentially methylated regions, which comprise approximately 0.06% of the human genome. This proportion is comparable to that of previous studies, although most such studies did not investigate proportions and used different methodologies^6,9,18^. Differential methylation was observed not only in promoter regions but also in intragenic and distal intergenic regions, with a higher proportion of hypomethylation than hypermethylation; this was consistent with the findings of a comparable MBD-seq-based study by Subhash *et al*.^16^ as well as a whole-genome bisulfite sequencing study by Kulis *et al*.^19^. While promoter hypermethylation is a well-known tumour suppressor mechanism, hypomethylation has not been as thoroughly investigated. Nevertheless, global hypomethylation (particularly in the intragenic regions) has been noted as a hallmark of CLL and presumed to contribute to genomic instability and gene activation during the pathogenesis of the disease^18^. Examples of hypomethylation in oncogenesis include (i) cancer-linked hypomethylation in gene regulatory regions^20,21^ and (ii) hypomethylation of interspersed and tandem repeats that promote tumour formation or progression by fostering DNA rearrangements^22,23^. It has also been shown that CpG islands that are not associated with the 5′ region but are located in intergenic or intragenic CpG islands of any gene can perform important biological functions^24^.

Noncoding genes that were frequently hyper- or hypomethylated accounted for up to 20% of DMGs, while the remaining 80% were protein-coding. DNA methylation is a relatively stable modification causing transcriptional inactivation of both protein coding genes and non-coding regulatory microRNA genes^25–27^ and is therefore a main mechanism of aberrant gene silencing in cancer^17^. The dysregulation of long noncoding RNAs such as long intronic noncoding RNAs promotes carcinogenesis, disease progression, and metastasis in various cancers^28,29^ including CLL^30^. These long noncoding RNA genes encode non-protein-coding transcripts of >200 nucleotides generated by RNA polymerase II, and their expression is tightly regulated in a cell type-specific and/or cellular differential stage-specific manner^31^. They comprised 12% of the DMGs in our study, and some were listed in the top 40 hypermethylated (including *LINC00273* and *LINC00839*) as well as in the top 40 hypomethylated genes (such as *LINC00348*); none of these three genes have been reported in previous Western studies. Meanwhile, microRNA genes, which encode a class of single-stranded noncoding RNAs 19–25 nucleotides in length^32^, can either be oncogenic or tumour suppressive^17^, and their aberrant methylation has clearly been implicated in CLL pathogenesis in previous studies^17,32^. Previously reported microRNA genes that elicit epigenetic changes in Caucasian CLL include *miR15a, miR16-1, miR-21, miR-29a, miR-34a, miR-139, miR-155, miR-574, miR-582*, and *miR1204*^17,32^. Aggressive and indolent CLLs exhibit a different microRNA profile, and high levels of miR-21 and miR-155 are associated with a greater mortality rate^32^. In our study, microRNA genes represented 5% of the DMGs, and four (*MIR4436A*, *MIR4537*, *MIR4715*, and *MIR7850*) that were among the top 40 hypermethylated genes have never been reported in previous Western studies. Further investigation on aberrant methylation of these novel long noncoding RNA and microRNA genes will provide a better insight into their roles in the pathogenesis of CLL in Korean individuals.

GO and pathway analyses showed findings consistent with those of previous CLL studies in Caucasians; the most significantly over-represented GO terms included those for lymphocyte differentiation, immune system development, lymphocyte activation, B-cell differentiation, and/or B-cell activation^17,18^. The roles of the B-cell receptor signalling pathway genes *SYK, PIK3AP1, PTPN6, MAP2K2*, and *NFATC1* in CLL pathogenesis have also been previously described^33–37^. *SYK* is a tyrosine kinase and is involved in the CD38 signal transduction pathway in CLL, and a selective Syk inhibitor is currently undergoing a clinical trial^34^. An expression study revealed that *PIK3AP1* is involved in the B-cell receptor signalling pathway in CLL as shown via functional enrichment analysis^37^. *PTPN6* encodes SHP-1 and is an important negative modulator of antigen-receptor signalling in lymphocytes; it is activated by *NOTCH1*, which has an important pathogenic role in CLL^33^. *MAP2K2* is involved in the RAS-BRAF-MAPK-ERK pathway, and mutations in this gene have been observed in CLL^34^. *NFATC1* activation by DNA hypomethylation in CLL correlates with clinical staging and can be inhibited by ibrutinib^36^. Our data demonstrated that CLL in Koreans shares common features with CLL in Caucasians in this regard.

Our network-based prioritization analysis identified genes that were differentially methylated and that are linked to many known CLL-related genes via protein-protein and protein-DNA interactions. Among the 10 hub DMGs revealed in our analysis, six (*STAT3, PTPN6, SYK, STAT5B*,and *XPO1*, and *ABL1*) had known CLL relevance; hence, our results corroborated previous data. The remaining four (*UBC, GRB2, CREBBP*, and *GAB2*) have never been reported in previous CLL studies^38–48^. *UBC* represents a ubiquitin gene (ubiquitin C) and has been described in cancers infrequently. In a previous study, interaction analysis of biomarker genes revealed that *UBC* may have a major role in renal cancer^38^. *GRB2* encodes growth factor receptor-bound protein 2 and has been described in cancers relatively frequently; as such, anti-cancer therapeutics targeting *GRB2* are currently in development^48^. *CREBBP* encodes chromatin-modifying enzymes such as the histone acetyl-transferases and has been studied in diffuse large B cell lymphoma, acute lymphoblastic leukaemia, and lung cancer^41–45^. *GAB2* encodes the *GRB2* associated binding protein 2^46^ and has been studied in breast cancer, ovarian cancer, hepatocellular carcinoma, lung cancer, and melanoma^47,48^. The top three genes most relevant to CLL in our network were *TP53*, *BCL2*, and *ZAP70*. Among them, *TP53* and *ZAP70* interacted with the four novel hub DMGs. The interactions of *UBC* and *CREBBP* with *TP53* represent post-translational regulation of the p53 protein via ubiquitination and acetylation^49^. *GRB2* and *GAB2* interacted with *ZAP70*^50^; the ZAP-70-mediated phosphorylation of the GRB2/GAB2 protein complex serves as a scaffold for the assembly of downstream signalling proteins^50^. Taken together, the interactions between the four hub DMGs and the well-known CLL-related genes underscore their biological significance.

Some recent epigenetic studies of CLL provided new insights into the chromatin landscape of this disease^19,51,52^; our data can be regarded as building on such knowledge. Previous studies that aimed to identify CLL-specific methylation events compared CLL cells to normal CD19+ B cells in order to pinpoint the specific features that represent the epigenetic characteristics of CLL^9,10,53,54^. Recent epigenetic studies also compared the chromatin landscape of CLL cells and B-cells from different maturation stages^19,51,52^ and observed that a large proportion of the differentially methylated sites overlap with those undergoing dynamic methylation during normal differentiation, mainly those of memory B-cells and bone marrow plasma cells^19,51,52^. This suggests that virtually all reported ‘CLL-specific’ differences reflected normal B cell maturation and are likely not causative of the disease^51^. They also reported that (i) early differentiation stages mainly displayed enhancer demethylation, which was associated with the upregulation of key B-cell transcription factors, and affected multiple genes involved in B-cell biology^19^; and (ii) CpGs losing methylation at any B-cell maturation stage were preferentially located in introns, intergenic regions, and repetitive elements^19^. As we did not compare the methylation patterns of our CLL cells to those of memory B-cells isolated from control individuals (as performed in previous Western studies), we infer that most of our differentially methylated regions (which were also identified in Western studies) might overlap with those of normal memory B-cells, and are maturation stage-specific rather than disease-specific. Nevertheless, our observation of over-represented B-cell receptor signalling pathway components and prevalent hypomethylation in the distal intergenic and intron regions were consistent with data from studies in which B-cells of different maturation stages were analysed separately, thereby affirming the credibility of our data. We infer that the common features shared with previous Western studies might have been derived from the chromatin landscape of normal memory B-cells, although the unique findings in our CLL samples (i.e., those which have not been reported in previous Western studies) differentiate Korean CLL from its Western counterpart. As Kulis *et al*. concluded, the changes shared during neoplastic transformation and normal differentiation may be epigenetic ‘passengers’, whereas those exclusively occurring in CLL cells, as we observed in our study, were likely epigenetic ‘drivers’ with a potential role in CLL development^19^.

A limitation of our approach is that we were unable to assess the expression statuses of genes speculated to be affected by differential hypo- or hypermethylation owing to the lack of RNA samples. Even though the main consequence of aberrant promoter methylation is dysregulated gene expression, the consequences of aberrant hypo- or hypermethylation are not limited to the alteration of transcription. Kulis *et al*. also reported that they rarely observed a direct correlation between gene expression and DNA methylation, even in regulatory elements, which was similar to previous observations^11,19,55,56^. In their study comparing *IGHV*-mutated and *IGHV*-unmutated CLLs, Beekman *et al*. suggested that the different cellular origins of these two types of CLL do not necessarily imply differential chromatin activation, likely owing to the fact that the differential DNA methylation in the *IGHV*-mutated and *IGHV*-unmutated CLL-originating cells is independent of the differential expression of the target genes^51^. In fact, dysregulated methylation outside the promoter can affect carcinogenesis and other conditions such as embryonic development, atherosclerosis, aging, and neural development via chromosomal instability^57^ or alternative splicing^58^. Thus, our methylation profiling data ought to be valuable even without considering gene expression, as they provide a comprehensive picture of aberrant hypo- and hypermethylation in Koreans with CLL.

In summary, we used MBD-seq to perform global methylation profiling of CLL in an Asian population for the first time. Our results showed that promoters were the preferential targets of differential methylation, as were distal intergenic and intron regions. Along with protein-coding genes, long intronic noncoding RNAs and microRNAs were frequently affected as well. Pathways related to immune processes and cancer were the main targets of aberrant methylation in Koreans with CLL, which is consistent with data from Caucasians. We also revealed novel candidate CLL-associated genes (*UBC*, *GRB2*, *CREBBP*, and *GAB2*) that closely interact with *TP53* and *ZAP70*, implying the existence of differences between CLLs afflicting Asians versus Caucasians.

## Methods

### Study populations

Eight ethnically Korean patients diagnosed with CLL without *IGHV* mutations between May 2008 and July 2014 at Hallym University Sacred Hospital, Republic of Korea, were enrolled. CLL was diagnosed based on the World Health Organisation^59,60^ and 2008 International Workshop on Chronic Lymphocytic Leukemia-National Cancer Institute criteria^61^. Collected laboratory data included complete blood counts, bone marrow pathology, immunophenotyping, conventional karyotyping, and *IGHV* somatic hypermutation status. Five age-matched, voluntary donors were examined as healthy controls. The study was performed according to the guidelines of the Declaration of Helsinki and was approved by the Ethics Committee of Hallym University (No. HALLYM 2019-01-004-002). All subjects provided written informed consent to participate in this study.

### MBD-seq library preparation and sequencing

Bone marrow buffy coats were collected from the patients; the median lymphoid cell percentage was 85.75% (range, 41.60–99.00%). CD19-positive B cells were collected from five healthy donors using magnetic bead sorting (EasySep^TM^; STEMCELL Technologies, Inc., Vancouver, Canada). Purity was confirmed using flow cytometry analysis (>95.0%). Genomic DNA was isolated using the Promega Maxwell® 16 MDx Instrument. Genomic DNA (1 µg) was sheared to 200–400 bp using a Covaris LE220 sonicator; the fragments were then subject to methyl-CpG enrichment using the Invitrogen MethylMiner Methylated DNA Enrichment Kit, which uses a recombinant form of human MBD protein-2. The enriched methylated DNA fragments were eluted as a single enriched population with a 2,000 mM NaCl elution buffer. The eluted DNA was then used to generate libraries according to the standard Illumina protocol. Briefly, the DNA fragments were subject to end repair, A-tailing of the 3′ end, Illumina adapter ligation, size selection (aiming for 300–500 bp), PCR amplification, and validation using an Agilent Bioanalyzer. The libraries were then sequenced on the Illumina HiSeq 2000 platforms.

### Pre-processing of sequencing data

FastQC was used to check the sequence quality of the 100 bp paired-end sequencing reads. Trimmomatic^62^ was used to clean the reads by removing adapter sequences, bases from the ends of the reads with quality <3, sliding windows of four bases with a mean quality <15, and reads shorter than 36 bp. The cleaned reads were aligned to the hg38 human genome with Bowtie2^63^ using default parameters. The resulting mapped data in BAM format served as an input to subsequent differential methylation analysis.

### Differential methylation analysis

The BAM files were inputted into the MEDIPS program^64^. The data for chromosomes 1–22 and M were selected for analysis. Sex chromosomes were excluded to avoid potential biases arising from X chromosome inactivation in samples from women. The genome was binned into adjacent windows 250 nucleotides in length. Differential methylation analysis between the CLL and control groups was performed at the window level. The parameter for data normalization and differential methylation analysis was set to edgeR and that for multiple testing correction was set according to the Benjamini-Hochberg procedure; default values were used for all other parameters. Windows with false discovery rates <0.01 were deemed differentially methylated. The methylation data are available at the Gene Expression Omnibus under the accession number GSE136986. The methylation levels of the differentially methylated windows were used for hierarchical clustering analysis to examine the separability of the CLL and normal sample groups. The methylation level of each window, as measured in log-counts per million values obtained from the edgeR R package^65^, was standardized using a z-transformation such that the row mean and variance were set to 0 and 1, respectively. Next, hierarchical clustering was performed in the EMA R package^66^ using the average linkage method with Pearson correlation analysis as the similarity metric.

### Annotation of the differentially methylated windows

The window-level differential methylation analysis result from MEDIPS was inputted into the ChIPseeker program^67^. For each window, ChIPseeker assigns a corresponding Entrez Gene and one or more of the following annotations: distal intergenic, promoter (2 kb upstream to 0.5 kb downstream of transcription start site), 5′ untranslated region, exon, intron, 3′ untranslated region, and downstream. Default values were used for all other parameters. After annotating the differentially methylated windows, the list of DMGs was obtained. A gene was deemed a DMG if at least one of the windows that corresponded to it was differentially methylated, although there was usually more than one; as such, the DMG’s p-value and log2 fold change were set to the smallest window-level p-value and the average of window-level log2 fold change values, respectively. The DMGs were classified with respect to gene type by the scheme adopted by ‘Ensembl’ into four categories: protein-coding, long noncoding, short noncoding, and others (pseudogenes and unannotated).

### GO and pathway analysis

Functional enrichment analysis was performed using the clusterProfiler program^68^ to identify prevalent biological themes in the DMG list using GO and KEGG pathway analyses, with significance set at a p-value < 0.05. For GO analysis, *minGSSize* = 20 and *maxGSSize* = 1,000 were applied. To compare the functional enrichment results between the directions of differential methylation and between genomic regions, a maximum of 30 GO terms or pathways were selected for each track, and their p-values were displayed side by side on a dot plot using the ‘clusterProfiler’ function. The KEGG^69^ pathway map for the B-cell receptor signalling pathway was rendered by the Pathview Web^70^.

### Network-based prioritization of DMGs

We examined how well the DMGs interact with known CLL-related genes (per the DisGeNET database)^71^ in human protein-protein and protein-DNA interaction networks obtained using Cytoscape^72^ and the associated applications BisoGenet^73^ and ReactomeFIViz^74^.

We first downloaded the list of CLL-related genes by searching the DisGeNET website using the keyword ‘chronic lymphocytic leukemia’; each of the CLL-related genes is annotated with supporting publications. From the obtained list, we removed genes that were annotated with only one publication to rule out potential false positives. Next, we constructed a network that links DMGs and known CLL-related genes; from BisoGenet, we retrieved the protein-protein interactions from all available sources as well as the protein-DNA interactions from the Biomolecular Interaction Network Database. We retrieved all available interactions from ReactomeFIViz, and excluded those that were only predicted. Gene symbols were used to query the interactions between genes in both applications; the two resultant networks were combined using Cytoscape’s ‘Merge’ function, and duplicate edges were then removed to yield a consolidated human interaction network. Node degree was obtained using Cytoscape’s ‘NetworkAnalyzer’ function^75^.

## Supplementary information

Supplementary information.

## Data Availability

The methylation data are available at the Gene Expression Omnibus under the accession number GSE136986.
