# Supplementary material for: Novel genes exhibiting DNA methylation alterations in Korean patients with chronic lymphocytic leukaemia: a methyl-CpG-binding domain sequencing study
Source: Sci Rep. 2020 Jan 23;10:1085. doi: 10.1038/s41598-020-57919-6 (PMC6978354; doi:10.1038/s41598-020-57919-6)

**Novel genes exhibiting DNA methylation alterations in Korean patients with chronic lymphocytic leukaemia: a methyl-CpG-binding domain sequencing study**

Miyoung Kim^1^, Eunyup Lee^1^, Dae Young Zang^2^, Hyo Jung Kim^2^, Ho Young Kim^2^, Boram Han^2^, Han-Sung Kim^1^, Hee Jung Kang^1^, Seungwoo Hwang^3*^, Young Kyung Lee^1*^

^1^Department of Laboratory Medicine, Hallym University Sacred Heart Hospital, Anyang, Republic of Korea; ^2^Department of Internal Medicine, Hallym University Sacred Heart Hospital, Anyang; Republic of Korea; ^3^Korean Bioinformation Center, Korea Research Institute of Bioscience and Biotechnology, Daejeon, Republic of Korea

*Correspondence:

Young Kyung Lee, M.D., PhD.

Department of Laboratory Medicine, Hallym University Sacred Heart Hospital

22, Gwanpyeong-ro 170 beon-gil, Dongan-gu, Anyang-si, Gyeonggi-do 14068, Republic of Korea

Tel: +82 31 380 3930, Fax: +82 31 380 3934, E-mail: [lyoungk@hallym.or.kr](mailto:lyoungk@hallym.or.kr)

Seungwoo Hwang, PhD.

Korean Bioinformation Center, Korea Research Institute of Bioscience and Biotechnology

125 Gwahak-ro, Yuseong-gu, Daejeon-si, 34141, Republic of Korea

Tel: +82 42 879 8544, Fax: +82 42 879 8519, E-mail: [swhwang@kribb.re.kr](mailto:swhwang@kribb.re.kr)

**Supplementary tables and figures**

**Supplementary Table 1. Top 40 hypermethylated genes with Entrez Gene ID annotations.**

Top 40 genes that were hypermethylated in the chronic lymphocytic leukaemia (CLL) group, and that have Entrez Gene ID annotations, compared to the normal group. Shading and numbers in the seven columns in the middle of the table indicate the presence and number of differentially methylated regions (DMRs) in each genomic annotation, respectively.

| Gene Symbol | Gene Name | Number of DMRs | | | | | | | Fold Change (CLL/Normal) | P-value |
| --- | --- | --- | --- | --- | --- | --- | --- | --- | --- | --- |
|  |  | Distal | Promoter | 5'UTR | Exon | Intron | 3'UTR | Downstream |  |  |
| ERBIN | erbb2 interacting protein |  |  |  |  | 3 |  |  | 14.41 Up | 5.21E-14 |
| LINC00273 | long intergenic non-protein coding RNA 273 | 9 |  |  |  |  |  |  | 8.76 Up | 1.80E-12 |
| AGBL5 | ATP/GTP binding protein like 5 |  | 2 |  |  |  |  |  | 35.63 Up | 2.32E-12 |
| MIR4537 | microRNA 4537 | 7 |  |  | 3 |  |  |  | 74.93 Up | 2.42E-12 |
| HCN1 | hyperpolarization activated cyclic nucleotide gated potassium channel 1 | 27 |  |  |  |  |  |  | 230.05 Up | 2.48E-12 |
| LHPP | phospholysine phosphohistidine inorganic pyrophosphate phosphatase |  |  |  |  | 5 |  |  | 47.02 Up | 3.40E-12 |
| KIF16B | kinesin family member 16B |  |  |  |  | 2 |  |  | 58.45 Up | 3.57E-12 |
| MYH3 | myosin heavy chain 3 |  |  |  |  | 1 |  |  | 33.31 Up | 7.26E-12 |
| LMNTD1 | lamin tail domain containing 1 |  |  |  |  | 2 |  |  | 14.28 Up | 9.13E-12 |
| CWH43 | cell wall biogenesis 43 C-terminal homolog | 139 |  |  |  |  |  |  | 9.93 Up | 1.51E-11 |
| TBC1D22A | TBC1 domain family member 22A |  |  |  |  | 4 |  |  | 27.28 Up | 2.22E-11 |
| SMIM11B | small integral membrane protein 11B | 6 |  |  |  |  |  |  | 5.62 Up | 3.02E-11 |
| FAM193A | family with sequence similarity 193 member A |  | 2 |  |  |  |  |  | 16.93 Up | 5.28E-11 |
| SNX29P2 | sorting nexin 29 pseudogene 2 |  |  |  |  | 2 |  |  | 14.63 Up | 9.34E-11 |
| TTC7A | tetratricopeptide repeat domain 7A |  |  |  |  | 1 |  |  | 18.54 Up | 1.46E-10 |
| LAMTOR5-AS1 | LAMTOR5 antisense RNA 1 |  |  |  |  | 2 |  |  | 24.39 Up | 1.80E-10 |
| DEFB115 | defensin beta 115 | 25 |  |  |  |  |  |  | 5.26 Up | 2.61E-10 |
| GNA12 | G protein subunit alpha 12 |  |  |  |  | 1 |  |  | 36.80 Up | 2.63E-10 |
| CCR6 | C-C motif chemokine receptor 6 |  | 1 |  |  | 3 |  |  | 30.96 Up | 2.89E-10 |
| CREBBP | CREB binding protein |  |  |  |  | 2 |  |  | 34.64 Up | 4.25E-10 |
| KCNJ18 | potassium voltage-gated channel subfamily J member 18 | 17 |  |  |  |  |  |  | 38.13 Up | 4.48E-10 |
| TPD52 | tumour protein D52 |  |  |  |  | 1 |  |  | 31.37 Up | 5.31E-10 |
| TCL1A | T-cell leukaemia/lymphoma 1A |  | 1 |  |  |  |  |  | 17.60 Up | 5.87E-10 |
| MIR7850 | microRNA 7850 |  |  |  |  | 2 |  |  | 27.67 Up | 6.22E-10 |
| MIR4715 | microRNA 4715 |  |  |  |  | 1 |  |  | 15.28 Up | 8.59E-10 |
| SNX16 | sorting nexin 16 |  | 2 |  |  |  |  |  | 25.89 Up | 1.22E-09 |
| EMB | embigin | 18 |  |  |  |  |  |  | 4.43 Up | 1.57E-09 |
| LINC00839 | long intergenic non-protein coding RNA 839 | 42 |  |  |  |  |  |  | 13.94 Up | 1.98E-09 |
| BANP | BTG3 associated nuclear protein |  |  |  |  | 3 |  |  | 20.73 Up | 2.02E-09 |
| SSPN | sarcospan |  |  |  |  | 2 |  |  | 78.03 Up | 2.06E-09 |
| NPIPB3 | nuclear pore complex interacting protein family member B3 | 2 |  |  |  |  |  |  | 22.52 Up | 2.18E-09 |
| TSSC1 | tumour suppressing subtransferable candidate 1 |  |  |  |  | 3 |  |  | 7.08 Up | 2.27E-09 |
| ZNF596 | zinc finger protein 596 | 2 |  |  |  |  |  |  | 8.59 Up | 3.04E-09 |
| SIDT2 | SID1 transmembrane family member 2 |  | 2 |  |  |  |  |  | 25.27 Up | 3.22E-09 |
| DTX1 | deltex E3 ubiquitin ligase 1 | 2 |  |  |  | 1 |  |  | 10.11 Up | 3.52E-09 |
| TMEM121 | transmembrane protein 121 | 2 |  |  |  |  |  |  | 9.36 Up | 3.69E-09 |
| MIR4436A | microRNA 4436a | 21 |  |  |  |  |  |  | 6.16 Up | 4.09E-09 |
| FAM81A | family with sequence similarity 81 member A |  |  |  |  | 1 |  |  | 23.83 Up | 4.53E-09 |
| MTMR10 | myotubularin related protein 10 |  |  |  |  | 1 |  |  | 16.20 Up | 4.71E-09 |
| SYPL1 | synaptophysin like 1 | 2 |  |  |  |  |  |  | 18.64 Up | 5.07E-09 |

**Supplementary Table 2. Top 40 hypomethylated genes with Entrez Gene ID annotation.**

Top 40 genes that were hypomethylated in chronic lymphocytic leukaemia (CLL), and that have Entrez Gene ID annotations, compared to the normal group. The table was prepared in the same manner as in Supplementary Table 1.

| Gene Symbol | Gene Name | Number of DMRs | | | | | | | Fold Change (CLL/Normal) | P-value |
| --- | --- | --- | --- | --- | --- | --- | --- | --- | --- | --- |
|  |  | Distal | Promoter | 5'UTR | Exon | Intron | 3'UTR | Downstream |  |  |
| RNA5-8SN5 | RNA, 5.8S ribosomal N5 |  |  |  |  | 6 |  | 1 | 30.16 Down | 3.72E-22 |
| SRGAP2-AS1 | SRGAP2 antisense RNA 1 | 269 |  |  |  |  |  |  | 49.41 Down | 9.71E-18 |
| SFTPB | surfactant protein B | 3 |  |  |  |  |  |  | 9.06 Down | 4.70E-16 |
| EPHA3 | EPH receptor A3 | 7 |  |  |  |  |  |  | 124.87 Down | 5.21E-16 |
| FBRSL1 | fibrosin like 1 | 1 | 1 |  |  | 5 |  |  | 86.41 Down | 5.40E-16 |
| ZNF609 | zinc finger protein 609 |  |  |  |  | 1 |  |  | 23.78 Down | 9.50E-16 |
| ITPK1 | inositol-tetrakisphosphate 1-kinase |  |  |  |  | 6 |  |  | 22.39 Down | 2.09E-15 |
| MIR4468 | microRNA 4468 | 1 |  |  |  |  |  |  | 57.36 Down | 2.38E-15 |
| LINC00348 | long intergenic non-protein coding RNA 348 |  |  |  |  | 1 |  |  | 32.84 Down | 3.75E-15 |
| KHDRBS3 | KH RNA binding domain containing, signal transduction associated 3 | 1 |  |  |  |  |  |  | 26.90 Down | 1.45E-14 |
| MUC2 | mucin 2, oligomeric mucus/gel-forming |  |  |  |  | 3 |  |  | 9.25 Down | 1.81E-14 |
| PACS2 | phosphofurin acidic cluster sorting protein 2 |  |  |  |  | 1 |  |  | 63.60 Down | 2.69E-14 |
| SLC22A12 | solute carrier family 22 member 12 |  |  |  |  |  | 2 |  | 11.83 Down | 4.56E-14 |
| ANKRD33 | ankyrin repeat domain 33 |  |  |  |  | 1 |  |  | 16.28 Down | 6.60E-14 |
| PCCA | propionyl-CoA carboxylase alpha subunit |  |  |  |  | 1 |  |  | 11.99 Down | 6.68E-14 |
| DLGAP2 | DLG associated protein 2 |  |  |  |  | 3 |  |  | 32.20 Down | 1.44E-13 |
| NEURL1 | neuralized E3 ubiquitin protein ligase 1 |  |  |  |  | 2 |  |  | 25.09 Down | 1.61E-13 |
| PTPRS | protein tyrosine phosphatase, receptor type S |  |  |  |  | 1 |  |  | 10.04 Down | 2.36E-13 |
| USP39 | ubiquitin specific peptidase 39 |  | 1 |  |  |  |  |  | 9.34 Down | 3.04E-13 |
| TMPRSS2 | transmembrane protease, serine 2 |  |  |  |  | 2 |  |  | 13.92 Down | 3.45E-13 |
| ZBTB17 | zinc finger and BTB domain containing 17 |  | 1 |  |  | 6 |  |  | 14.19 Down | 3.76E-13 |
| RNA28SN5 | RNA, 28S ribosomal N5 |  | 3 |  |  |  |  |  | 8.70 Down | 4.20E-13 |
| UQCRFS1 | ubiquinol-cytochrome c reductase, Rieske iron-sulfur polypeptide 1 | 3 |  |  |  |  |  |  | 14.64 Down | 4.71E-13 |
| ADCY3 | adenylate cyclase 3 |  | 1 |  |  |  |  |  | 21.53 Down | 6.77E-13 |
| BMP7 | bone morphogenetic protein 7 |  |  |  |  | 2 |  |  | 41.21 Down | 6.85E-13 |
| SLC45A4 | solute carrier family 45 member 4 |  |  |  |  | 2 |  |  | 9.07 Down | 1.08E-12 |
| CAMSAP3 | calmodulin regulated spectrin associated protein family member 3 |  | 1 |  |  | 4 |  |  | 88.27 Down | 1.82E-12 |
| AKR1B1 | aldo-keto reductase family 1 member B | 2 |  |  |  |  |  |  | 6.85 Down | 2.16E-12 |
| TRAF7 | TNF receptor associated factor 7 |  |  |  |  | 1 |  |  | 12.40 Down | 2.19E-12 |
| SYNPO | synaptopodin |  | 2 |  |  |  |  |  | 14.03 Down | 3.09E-12 |
| HDAC5 | histone deacetylase 5 |  | 1 |  |  |  |  |  | 7.79 Down | 3.35E-12 |
| ELOVL6 | ELOVL fatty acid elongase 6 |  |  |  |  | 1 |  |  | 13.97 Down | 3.61E-12 |
| EHD1 | EH domain containing 1 |  |  |  | 1 |  |  |  | 6.78 Down | 3.77E-12 |
| SLC7A7 | solute carrier family 7 member 7 |  |  |  |  | 3 |  |  | 9.52 Down | 3.99E-12 |
| MIR662 | microRNA 662 |  | 2 |  |  |  |  |  | 12.88 Down | 4.18E-12 |
| EMB | embigin | 25 |  |  |  |  |  |  | 104.59 Down | 4.21E-12 |
| PPP2R3A | protein phosphatase 2 regulatory subunit B''alpha | 1 |  |  |  |  |  |  | 22.03 Down | 5.53E-12 |
| ABCA7 | ATP binding cassette subfamily A member 7 |  |  |  |  | 2 |  |  | 21.64 Down | 5.96E-12 |
| FCGR2A | Fc fragment of IgG receptor IIa | 1 |  |  |  |  |  |  | 15.03 Down | 6.44E-12 |
| ZNF775 | zinc finger protein 775 |  | 2 |  |  |  |  |  | 8.19 Down | 6.88E-12 |

**Supplementary Fig 1. Most significantly over-represented Gene Ontology (GO) terms, analysed with respect to genomic annotation.** A maximum of 30 GO terms were selected for each track; the dot plot shows their p-values (see colour code legend). The statistical significance of a GO term increases with a redder tint. The number of differentially methylated genes belonging to a GO term increases with its size. The absence of a dot denotes insignificant over-representation of the corresponding GO terms in that particular condition. GO terms directly related to immune processes and cancer are shown in bold-type.

**
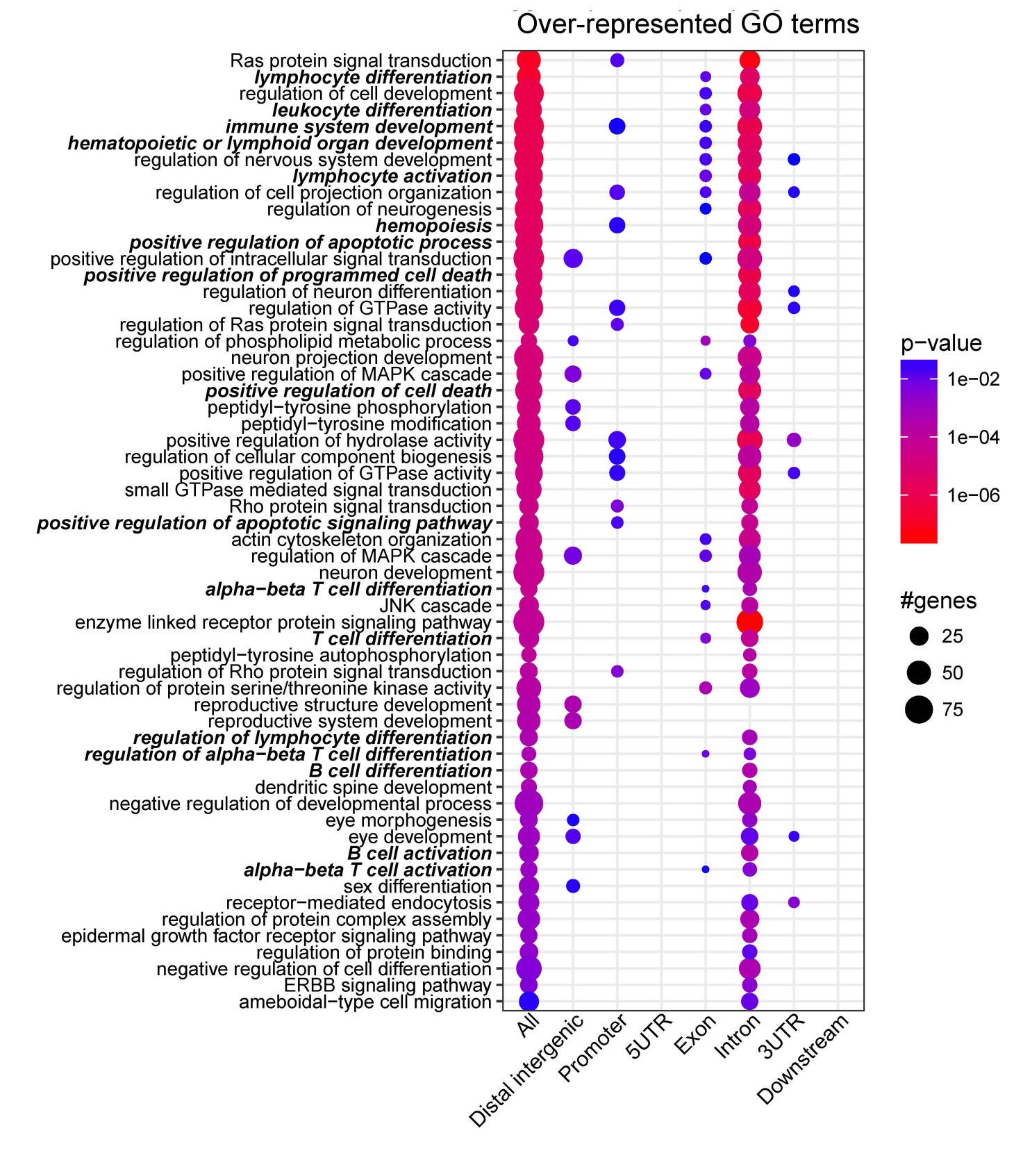
**

**Supplementary Fig 2. Most significantly over-represented pathways, analysed with respect to genomic annotation.** The plot was prepared in the same manner as in Supplementary Fig 1.


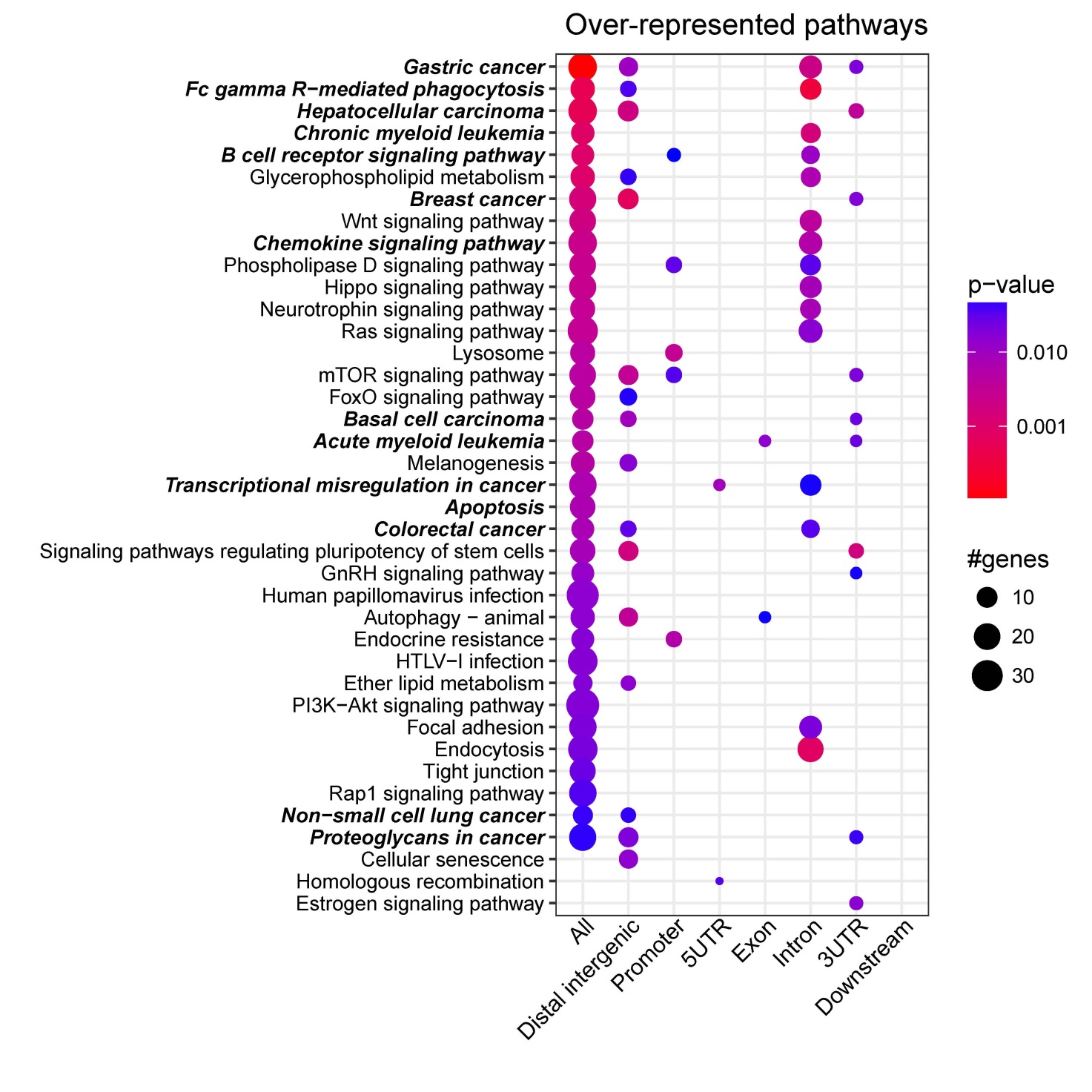

Supplement: Supplementary file 1 — Supplementary information. [file 41598_2020_57919_MOESM1_ESM.docx]
